# Clinical-scientist-led transoesophageal echocardiography (TOE): using extended roles to improve the service

**DOI:** 10.1136/bmjoq-2023-002268

**Published:** 2023-09-25

**Authors:** Nikki Kaye, Michael Purdon, Rebecca Schofield, Grazia Antonacci, Nathan Proudlove

**Affiliations:** 1 North West Anglia NHS Foundation Trust, Peterborough, UK; 2 Department of Primary Care and Public Health, NIHR ARC NWL, Imperial College London, London, UK; 3 Business School, Centre for Health Economics and Policy Innovation (CHEPI), Imperial College London, London, UK; 4 Alliance Manchester Business School, The University of Manchester, Manchester, UK

**Keywords:** Quality improvement, Statistical process control, Quality improvement methodologies, Continuous quality improvement

## Abstract

At the North West Anglia NHS Foundation Trust, we perform transoesophageal echocardiography (TOE), a semi-invasive diagnostic test using ultrasound for high-quality heart imaging. TOE allows accurate diagnosis of serious heart problems to support high-quality clinical decision-making about treatment pathways. The procedure can be lengthy and is traditionally performed by a consultant cardiologist, who typically has multiple commitments. This constrains patient access to TOE, leading to waits from referral to test, delaying treatment decisions.

In this quality improvement project, we improved access by redesigning workforce roles. The clinical scientist, who had been supporting the consultant during TOE clinics, took on performing the procedure as the main operator. We used the Model for Improvement to develop this clinical-scientist-led service-delivery model, and then test and refine it. This increased capacity and frequency of TOE clinics, reducing waits and releasing around 2 days per month of consultant time.

Over five plan-do-study-act cycles, we tested six changes/refinements. Our targets were to reduce the maximum waiting time for TOE to 3 working days for inpatients and to 14 working days for outpatients. We succeeded, achieving reductions in mean waiting times from 7.7 days to 3.0 days for inpatients and from 33.2 days to 8.3 days for outpatients.

TOE requires intubation; when this fails, TOE is abandoned. We believe light (rather than heavy) sedation is helpful for this intubation. We reduced sedation levels (from a median of 3 mg of midazolam to 1.5 mg) and, as a secondary outcome of this project, reduced the intubation failure rate from 13% to 0% (over 32 postchange patients).

Following this project, our TOE service is usually performed by a clinical scientist in echocardiography who has British Society of Echocardiography TOE accreditation and advanced training. We have sustained the improved performance and demonstrated the value of enhanced roles for clinical scientists.

**WHAT IS ALREADY KNOWN ON THIS TOPIC**

- Consultant cardiologist capacity in the English National Health Service (NHS) is severely constrained, limiting access to diagnostic procedures such as transoesophageal echocardiography (TOE) for both inpatients and outpatients; this has not been investigated in the literature.

**WHAT THIS STUDY ADDS**

- This study demonstrates how a TOE service can be redesigned for clinical scientist leadership and achieve results as good as (or better than) under a consultant cardiologist, improving patient access and liberating consultant cardiologist time.

**HOW THIS STUDY MIGHT AFFECT RESEARCH, PRACTICE OR POLICY**

- This paper suggests that a scientist-led service could become the norm for TOE and demonstrates a practical approach for other organisations to set up such a service safely.
- More generally, this study highlights the potential and importance of developing the roles of clinical scientists and how they can relieve consultant-grade doctors of some tasks.

## Problem

Echocardiography is an important diagnostic technique with high demand. Of the 15 key diagnostic tests monitored in public NHS England data, the echocardiography waiting list is the fourth largest and has the third highest number of 6-week wait breaches, which is the joint highest proportion at 48%.^1^ The national target set in 2008 is 1%. As of the end of September 2022, North West Anglia NHS Foundation Trust (NWAFT) had a waiting list of over 2800 for *echocardiography* (most being transthoracic echocardiography (TTE), but also *transoesophageal echocardiography* (TOE)) with 69% waiting over 6 weeks.

**Figure 1:**
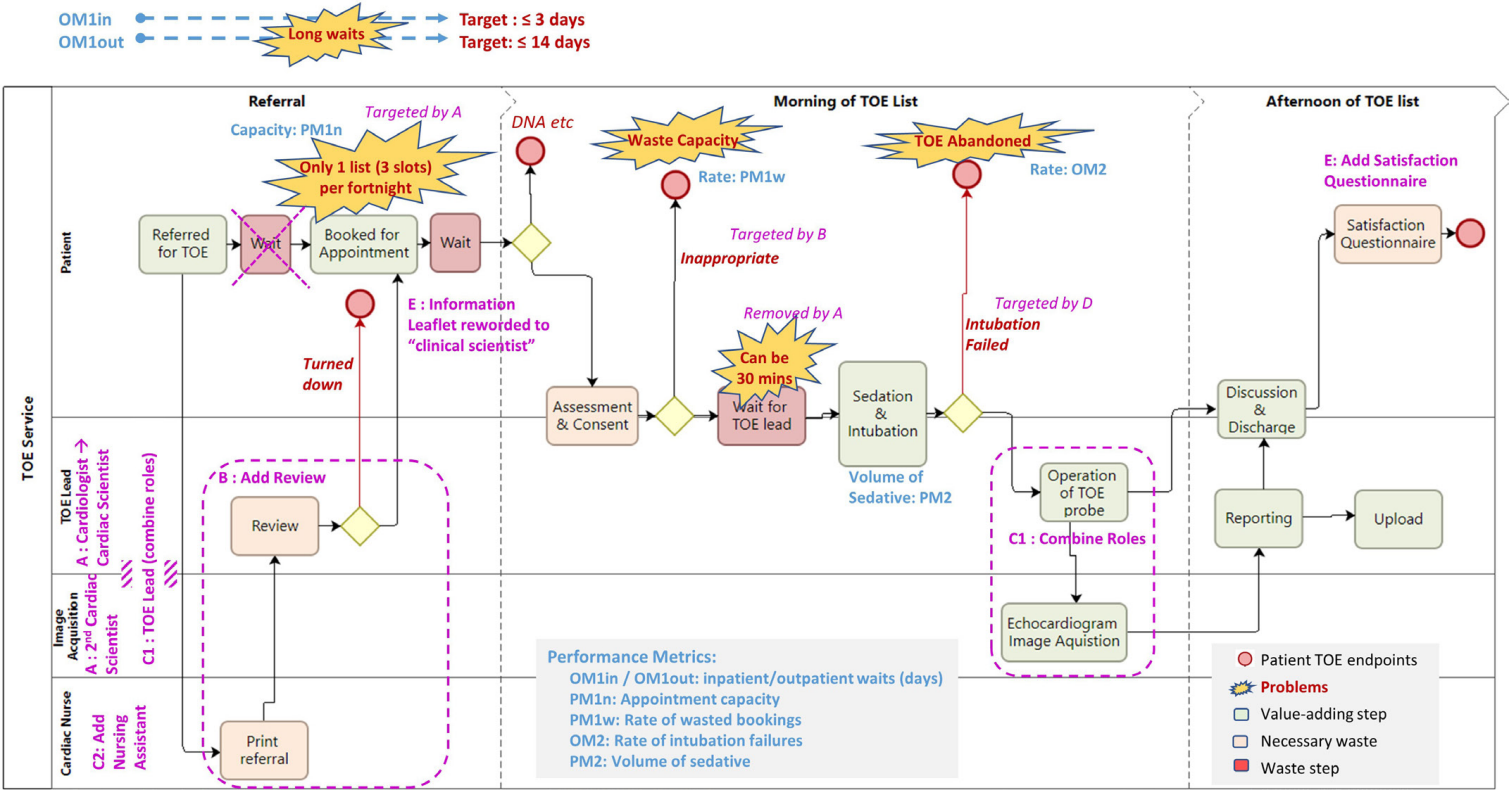
TOE service process map. BM, balancing metric; DNA, did not attend; OM, outcome metric; PM, process metric; PDSA, plan-do-study-act cycle; TOE, transoesophageal echocardiography; TTE, transthoracic echocardiography. The letter codes refer to the change ideas.

At the NWAFT Cardiac Investigations Department, TOE has been performed by a consultant cardiologist, a role in short supply and with multiple other commitments, so capacity has been limited. Additionally, sometimes TOE had to be abandoned due to failed intubation, and some appointment slots have been taken up with ‘failure demand’^2^ from inappropriate referrals. The pathway also contained many points of delay (figure 1), causing knock-on effects in other clinics and staffing.

Constrained access has lengthened patient waiting times for TOE, delaying clinical decision-making and so treatment pathways and condition management. For inpatients, it increased their length of stay in beds in our trust’s acute (secondary care) hospitals when patient flow is a particularly severe problem across the NHS.

Most inpatient referrals for TOE are ‘query infective endocarditis’ (IE), a condition that should be treated without delay to improve survival. Supporting this, an internal audit of IE at NWAFT found that long waits between inpatient referral for TOE and the TOE procedure itself (and so diagnosis) can affect patients’ prognoses. A further benefit of improved access to TOE would be some patients bypassing the inpatient TTE diagnostic service, saving departmental resources. TTE is a much higher-volume service, so an inpatient could access this more quickly. Therefore, in practice, clinicians may request a TTE even though they believe a TOE will be probably be necessary subsequently.

NWAFT’s vision is based on ‘excellent quality of care’ and ‘delivering outstanding care and experience’.^3^ We operationalise this through continuous service improvement and redesign initiatives, in particular, new quality improvement (QI) programmes.^3^

In this project, our QI approach was the Model for Improvement (MfI). At its core it has three questions: Q1: “What are we trying to accomplish?”; Q2: “How will we know that a change is an improvement?”; and Q3: “What changes can we make that will result in improvement?” to guide system exploration and plan-do-study-act (PDSA) cycles.^4^ The three questions guide a QI team to set aims, establish metrics and design and select change ideas to then test and refine through PDSA cycles. Revisiting the purpose, metrics and set of change ideas, the team decides whether to continue developing and testing further change ideas or to focus elsewhere. Recently, the MfI has been used successfully in another area of echocardiography,^5^ another physiological sciences specialism, neurophysiology,^6^ as well as in hospital life sciences specialisms.^7–9^

Our primary project aim (Q1 of the MfI) was to improve the performance of our department at NWAFT via improving our TOE provision. Within this overall aim, we had four goals (see also the driver diagram^4^ in online supplemental figure S3):

10.1136/bmjoq-2023-002268.supp4 Supplementary data

1. Improve access to TOE by reducing the waiting times to within 3 days for inpatients and 14 days for outpatients, within 6–12 months (our main intended outcome);
2. Improve intubation success rates, so fewer TOE procedures are abandoned;
3. Relieve the consultant cardiologist of the TOE clinic, so they can shift their time to other high-value tasks such as seeing patients who require clinical decisions, and to make use of the greater availability of clinical scientists;
4. Improve the quality of TOE reporting to meet British Society of Echocardiography (BSE) TOE guidelines.^10^

To try to achieve these, we tested a set of change ideas (Q3 of the MfI) arising from the broad concept of developing and refining a clinical-scientist-led TOE service. To our knowledge, there has been no previous performance analysis of a TOE service, or comparison of consultant- versus scientist-led provision, in the NHS. Therefore, if successful, an additional objective was to disseminate our findings. To assess our progress (Q2 of the MfI), we established and analysed a set of metrics. See online supplemental figure S3 for an overview of the logic of our project.

## Background

TOE is a semi-invasive diagnostic test using ultrasound to produce high-quality images of the heart. Unlike in TTE, in TOE the echo transducer that produces the sound waves is attached to a soft, thin, flexible probe inserted through the patient’s mouth and down the oesophagus. This is an uncomfortable procedure and we recommend patients are sedated, usually with midazolam, though they can choose not to be. Ideally, patients are *lightly* sedated, so they are less uncomfortable but still conscious enough to help with intubation by swallowing. Further sedation can be given if necessary. The relationship between the amount of sedation given during TOE and intubation success is a topic of considerable current interest in the field, but we could find no published papers.

The oesophagus lies close to the upper chambers of the heart and enables the operator to obtain clearer images of the heart and valve structures. TOE allows accurate diagnosis of serious heart problems, important for appropriate treatment and management. TOE is often used to provide information prior to heart surgery, for example, to repair or replace heart valves, and specialist regional (tertiary care) hospitals require TOE images for some patients before they accept their transfer. Some other patients are referred for TTE, but then the operator is unable to image the heart sufficiently clearly, and so they are referred-on for TOE.

Traditionally, the lead operator undertaking TOE has been a clinician, often a consultant cardiologist—an expensive and severely constrained resource in the NHS—as has been the case at NWAFT. However, there is increasing appreciation of the potential value and professionalism of clinical cardiac scientists, and the profession has grown considerably over the past few years, with increased responsibility and workload. Roles are widening, and the number of scientist-led services in echocardiography is growing, including heart valve clinics, stress echocardiography, contrast/bubble echocardiography clinics and (less commonly) TOE services.^11^

The NHS faces severe staffing shortages; as many as 10% of posts are unfilled in some roles and regions.^12 13^ The GIRFT (Getting It Right First Time) report for cardiology highlights that this shortage also extends to consultant cardiologists and cardiac clinical scientists.^14^ The report suggests that there should be an Urgent 7/7 TOE service with the support of a nurse. Our trust currently cannot provide this. It also suggests that achieving this coverage requires a national programme to expand the echocardiography workforce. Responding to these pressures, there is a pathway for cardiac physiologists to become clinical scientists and so able to register with the Health and Care Professions Council. This is via MSc-level Scientist Training Programme training positions or equivalence through the Academy for Healthcare Science.

GIRFT also suggests innovative use of the existing workforce, such as expanding the roles of cardiac clinical scientists.^14^ The report highlights successful examples of efficiency gains through switching to non-medically led services, with a consultant cardiologist available to support as needed. To take over such roles from consultants, clinical scientists must undergo advanced training. The National School for Healthcare Science has a new 18-month programme, the Echo Training Programme, to increase the number of echocardiographers by enabling cardiac scientists/physiologists to widen their roles, for example, performing more TTE and freeing some clinical scientists to do more specialised work like TOE.

A scientist-led service in TOE is not novel—a number of other NHS hospitals in the UK have instituted this approach. Our informal discussions with cardiac scientists from other trusts suggest these services operate successfully, but that training and standard operating procedures differ somewhat. There is no standardised training protocol for TOE advanced training (we suggest an outline of training for clinical scientists in TOE in online supplemental appendix A). Our literature search found no published studies or data on clinical-scientist-led TOE services.

10.1136/bmjoq-2023-002268.supp1 Supplementary data

Within echocardiography services, scientist-led TOE is less established than in other areas such as stress echocardiography or specialist valve clinics. This because TOE is semi-invasive and so carries greater risk. While clinical scientists in cardiac science perform some fully invasive techniques (such as implantation of loop recorder monitoring), scientist-led TOE requires simultaneous responsibility for the patient’s sedation and for analysis and interpretation of the results.

Staff retention is also challenging. In addition to workload pressures, cardiac clinical scientists have been demotivated by limited further career development, progression, research and education opportunities; this has led to an increasing number leaving the NHS to work through agencies or for private-provider companies.^11^ One recognised way to improve motivation and retain staff is to extend scientists’ responsibilities through the development of cardiac-scientist-led clinics, as reported to have been done to a greater extent in other echocardiography services.^11^ Thus, we believe extending this through TOE services could contribute to the retention of scientific staff in the NHS.

There has been a little use of QI in cardiac science more widely, for example, increasing activity in transthoracic echocardiography,^5^ reducing inappropriate echocardiography in paediatrics,^15^ patient/carer compliance with follow-up after fitting an implantable device also in paediatrics,^16^ increasing efficiency in cardiac catheterisation^17 18^ and improving process of care and outcomes generally in a developing country.^19^

## Measurement

The process flow for the service is outlined using process mapping^20–23^ in figure 1. Prior to the project, the consultant cardiologist operated the probe in the patient’s oesophagus and I (lead author, NK, cardiac clinical scientist) supported acquiring images on the echocardiography machine. We were supported by a cardiac nurse.

During the COVID-19 pandemic, the TOE service (classed as an aerosol-generating procedure) was temporarily suspended. Once we restarted, it took a while for the system to return to regular pre-COVID-19 operation. It had done so by summer 2021, and we used data from July 2021 to March 2022 for our baseline and root cause analyses.

**Figure 2:**
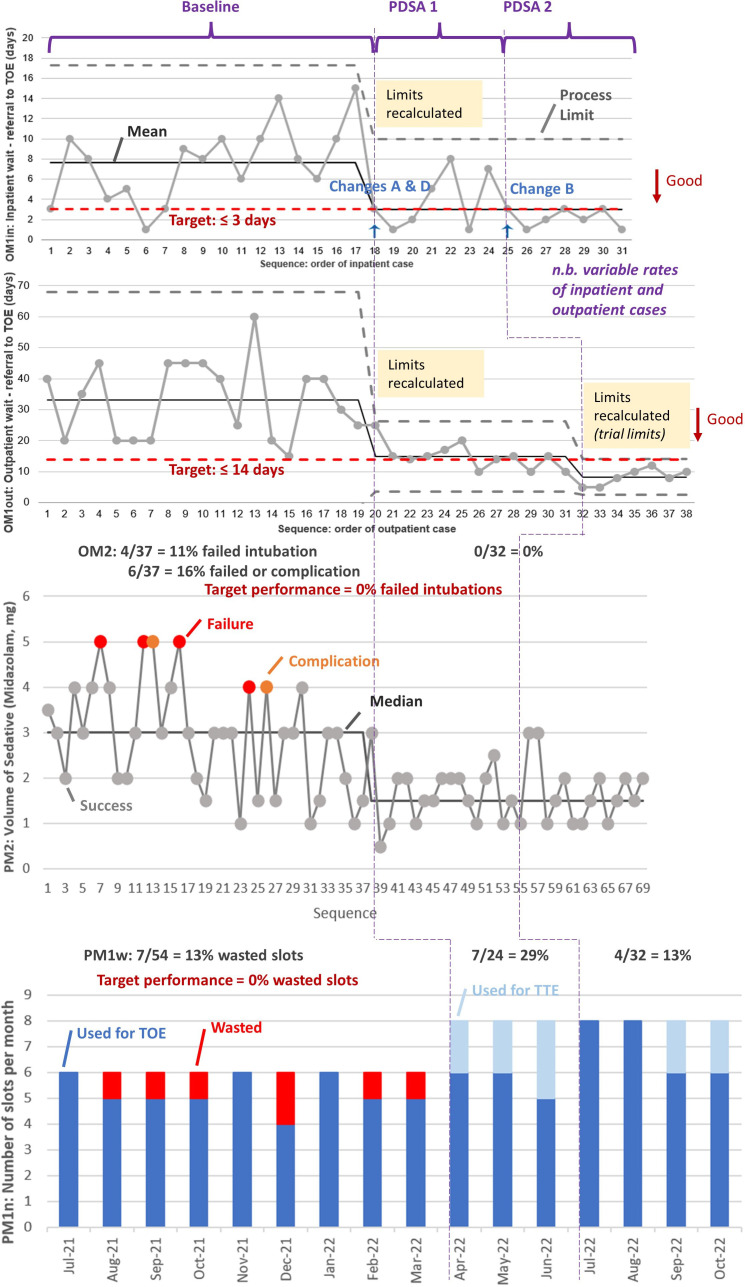
Metrics over time. PDSAs 3–5 not shown as not targeting these metrics. BM, balancing metric; OM, outcome metric; PM, process metric; PDSA, plan-do-study-act cycle; TOE, transoesophageal echocardiography; TTE, transthoracic echocardiography.

For goal 1, reducing waits for TOE, we set up two outcome metrics (OMs): OM1in, the time from referral to TOE for inpatients; and OM1out, the same for outpatients (figure 1). Figure 2 shows these in individuals (I or X) statistical process control (SPC) format.^24 25^ Baseline data show mean waits of 7.7 and 33.2 working days (Monday–Friday), respectively, well beyond our self-set targets of 3 and 14 days and with considerable variation. Useful process metrics (PMs) here were PM1n, the number of slots we could schedule for TOEs (capacity), and PM1w, the proportion of these not used for TOE (‘wasted’). The baseline values were PM1n=6 per month (fortnightly lists of 3 TOE slots each), and PM1w=7/54=13% wasted. Waste slots include patient did-not-attend (DNA), but also bookings turning out to be for inappropriate referrals, discovered through review on the day of the procedure (so too late to backfill). However, the reasons for unused slots were not documented, so we were unable to break this down retrospectively.

Goal 2 was to improve the success rate of intubation (OM2), with postulated drivers being the amount of sedation (midazolam) administered (PM2) and the operator (see online supplemental figure S3). Figure 2 shows PM2, in run chart format^25^ with baseline median=3 mg (mean=2.92 mg), and picks out cases where the patient struggled to tolerate the probe leading to intubation failure (TOE abandoned) or complications (taking extra time and often requiring additional medication such as pethidine). As shown, the baseline intubation failure rate (OM2) was 4/36=11%; adding complications takes this to 17%.

Goal 3 was to free consultant cardiologist time by releasing them from the TOE service. We can estimate the hours consumed. A TOE procedure usually takes around 60 min, including patient preparation (cannulation, consent, checking previous history). Later, time is required for discussing the results with the patient, results reporting and patient discharge. A morning session would typically run 09:00–12:00, that is, 3 hours, but can overrun. The cardiologist would then typically spend the afternoon analysing the cases, reporting, letter-writing, discussing with patients and discharging them—another 3 hours. Therefore, substituting with a clinical cardiac scientist as the main procedure-operator for a list would release approximately a day of consultant cardiologist time. As per PM1n, with the consultant-led service, we were scheduling two lists each month, thus consuming around 2 days of consultant cardiologist time per month.

Goal 4 was to improve reporting. We found that outpatients’ results were reported in a letter, while inpatients’ results were written in the patient’s notes. Examining our baseline data revealed that there was no standardisation in reporting; fewer than half of our TOE investigations were fully documented on the reporting system, meaning other healthcare professionals could not access analysis and results for some patients. No audit or quality assurance system was in place.

I (lead author, NK), as the person most involved with TOE, used a fishbone diagram^26^ to capture my perceptions on potential root causes of the problem goals 1, 2 and 4 aimed to address (long patient waits, intubation failures and less-than-idea reporting), see online supplemental figure S1. A 4N chart and niggle-o-gram^27–29^ captures and ranks perceptions gathered from a focus group of clinical scientists and cardiac nurses in the department, see online supplemental figure S2 and table S1. The top ‘niggles’ (from clinical scientists and cardiac nurses) were about long patient waits and access (staff were very aware of the long waits for TOE and TTE, NWAFT figures were noted at the start of this paper), with frustration with inefficiencies in the TOE process flows.

To give an overview of the resulting cause–effect relationships hypothesised, we developed a driver diagram,^30^ see online supplemental figure S3. This ‘causal map’ naturally was refined during the project, with C1 and C2 being refinements from the basic idea (A) of the scientist taking over operation of the TOE probe. When we changed the patient information leaflet (E), we took the opportunity to include a patient satisfaction questionnaire to check for adverse impacts of the change on patient experience, and to look out for any other potential further changes. We also considered potential barriers and actions that might help overcome them (online supplemental table S2).

## Design

This QI project was conducted by a small team consisting of the service manager, consultant cardiologist and cardiac nurse, led by the first author (NK, a clinical cardiac scientist). The team worked closely to enable me to collect the data. All reviewed the baseline data and analyses (figures 1 and 2 and online supplemental file 4).

Reviewing the process map, SPCs and fishbone diagram, it was apparent that there was waste capacity from slots cancelled on the day when a booking was found to be an inappropriate referral. On-the-day cancellations can be confusing and distressing for patients.^31^ We also had some lists with two outpatient TOEs plus one reserved for potential inpatient requests, with the latter sometimes not needed, and also some wasted time in the workflow (figure 1)—which can result in unnecessary list overruns.

The team identified six change ideas (A–E). See also the driver diagram (online supplemental figure S3). (Note: the initial idea driving the project was A; the others came up during the project as opportunities from, or refinements to, the initial idea. They are discussed in the order in which they are labelled on online supplemental figure S3.)

### Change idea A

A clinical cardiac-scientist replaces the consultant cardiologist as lead for the service and TOE probe operator during sessions.

Frequently, there were long waits for the cardiologist (much in demand for other tasks) (figure 1). Their availability also restricted the service to run a list only fortnightly. If a slot was unused, then that capacity would be wasted.

The core improvement idea was to release the consultant cardiologist with me (NK) stepping-up to be lead operator in their place. I already had the necessary training and experience (see online supplemental appendix A for notes on TOE training and accreditation). Clinical support is available onsite for any serious complication. The cardiac nurse was also deemed competent in delivering sedation through their experience and training. For their further development, the cardiac nurse will be present during the consent process and discussion of the results. To replace me in acquiring the echocardiography images, we would need a second clinical scientist/cardiac physiologist.

We predicted this would have a positive effect on the TOE service in many ways (online supplemental figure S3). Though it would put more pressure on the department by pulling in a second scientist/physiologist, this would be outweighed by the liberated high-value consultant cardiologist time.

No longer being constrained by cardiologist availability, we could run weekly lists, increasing capacity from 6 TOEs per month to 12 (3 TOEs per list), though initially limiting lists to 2 TOEs (so 8 per month) as we develop the service while ensuring patient safety. We aim to perform 12 per month once the consultant cardiologist and the cardiac scientist are satisfied. The greater capacity and shorter gaps between lists should reduce waits. A further advantage of having a scientist lead is that any unused TOE-list time (which was wasted previously) could be used for inpatient TTEs (so these could be done more quickly).

### Change idea B

Clinical scientist review of TOE referrals before booking.

Previously, the cardiac nurse would book *all* patients referred to the TOE service. There is patient risk associated with TOE, so it is important for patients to only have the investigation when required. On taking over the service (idea A), I found TOE was not appropriate for several patients arriving for appointments, so I cancelled them, which is poor patient experience and left unused TOE slots, or I performed TTE on them instead.

The change idea was for the cardiac nurse to print off referrals for my review *before* booking. This would increase activity (used slots), reduce unnecessary risk to patients and also allow me to prioritise inpatients (who are more urgent).

### Change idea C1

Release the second cardiac clinical scientist.

After taking on the lead role (idea A), I realised we could rearrange the room, putting the echocardiography machine on the same side as the TOE equipment, so I could operate both, without this compromising performance. We could therefore refine idea A, freeing the second scientist for other services—for example, instead of being present for a TOE list they could run a TTE list (approximately five procedures).

I predicted that this would not affect TOE performance metrics. TOE is a core part of my expertise and training, and performing both roles (manipulating the TOE probe and acquiring the images on the echocardiograph) is demanding but feasible, and would be efficient as I know exactly what images I wish to acquire.

### Change idea C2

Use a nursing assistant.

The above refinement resulted in only two of us in the room for sessions, which we found challenging for patient management. So a further refinement was to obtain support from a nursing assistant during the TOE procedure. (The nursing assistant already performed preprocedure checks at check-in such as ECG, blood pressure, oxygen saturation.)

### Change idea D

Reduce patient sedation.

As noted in the Background section, there is interest among TOE specialists in the impact of the degree of patient sedation on the success of intubation (and so of the whole procedure). We shared the view that light sedation tends to be more helpful than heavy. Taking over the service gave me and the cardiac nurse the opportunity to put this into practice.

### Change idea E

Change patient information leaflet and include satisfaction form.

The patient information leaflets stated that the procedure would be performed by a doctor (consultant cardiologist); we should change this to ‘by a senior clinical scientist in echocardiography or a consultant cardiologist’ to inform them, in advance of consent and to avoid creating confusion or anxiety. To investigate whether the new process was producing poor patient experience, we could also include a patient satisfaction survey and also take the opportunity to ask for experience-improvement suggestions.

The economic impact of the project was not a material consideration in the change and we did not model or evaluate this. However, the difference between the initial service and the final design (from a consultant + a clinical scientist, to a clinical scientist + a nursing assistant) is clearly substantial. A back-of-the envelope analysis suggests net salary savings of the order of 40%. The future trajectory is for the cardiac scientist specialising in TOE to become a consultant cardiac scientist (this is the purpose of the national Higher Specialist Scientist Training (HSST) programme). With this eventual salary uplift, we estimate the net salary savings would be of the order of 25%.

## Strategy

Over the course of this QI project, we tested and refined the change ideas over five PDSA cycles, summarised in table 1.

**Table 1:** PDSA improvement cycles

PDSA cycle	Plan/prediction	Do	Study	Act	Time required (months)
Baseline			OM1in: 7.7 days; OM1out: 33.2 days ☒ OM2: 11% intubation failures ☒ PM1n: 6 TOE slots per month PM1w: 13% slots wasted ☒ PM2: 2.92 mg of midazolam (median 3 mg)		
1	Institute a clinical scientist led service, extending role (CI A) and reduce sedation (CI D). This will improve OM1 (reduced waiting times due to more capacity, PM1n) and OM2 (greater intubation success rate).	Clinical cardiac scientist as lead operator 2nd cardiac scientist/physiologist in support (image acquisition) Weekly TOE lists (capacity 6 → 8 TOEs per month) Unused slots → TTE cardiac nurse delivers reduced sedation (midazolam).	OM1in: 3.9 days; OM1out: 15.0 days ☒ but successful reduction OM2: 0% intubation failures ☑ PM1n: 8 TOE slots per month ☑ PM1w: 29% slots wasted ☒ Unsuccessful—worse! (*but* can use for TTE) PM2: 1.66 mg of midazolam (median 1.5 mg) ☑	Do not recalculate the SPC limits for OM1in; recalculate for OM1out. Worthwhile improvement, make permanent change: Capacity=8 TOEs per month; Cardiologist 2 days a month released; Continue lower sedative. Inappropriate bookings: *institute referral review*	3
2	Institute review of referrals prior to booking and give priority to inpatients (CI B). This will further improve OM1 (by reducing slots wasted, PM1w, by reducing inappropriate bookings).	Cardiac nurse prints referrals. Reviewed by clinical scientist Appropriate cases booked, prioritising inpatients	OM1in: 2.1 days; OM1out: 8.3 days ☑ OM2: 0% intubation failures ☑ PM1w: 13% slots wasted ☒ (but used for TTE)	Recalculate SPC for OM1in, (at PDSA1). Provisional recalculation for OM1out at PDSA2. Worthwhile improvement, make permanent change. Continuing problem with end of week referrals—unable to review—refine in future.	2
3	Reassign roles to avoid need for a support (2nd) clinical scientist (CI C1). Can be done without reducing performance Frees resource for other departmental services.	Clinical scientist (lead operator) and cardiac nurse share image acquisition. Do not require a support clinical scientist.	No change to performance metrics (success!)	Release 2nd clinical scientist from here on. Technical success, but care management is difficult with challenging patients: *refine*	2
4	Add nursing assistant role to help with manipulation of patient’s head and ensure safety is maintained (CI C2). Easier management, especially of challenging patients.	Add nursing assistant role.	Easier patient management—successful	Worthwhile improvement, additional role justified, retain as permanent change.	3
5	Add ‘clinical scientist’ as TOE operator on patient information leaflet and institute patient satisfaction form to assess experience and gather patient suggestions (CI E). Patients will be reassured.	Discuss with stakeholders. Change the information leaflet. Include a patient satisfaction form with appointment letter.	No change to performance metrics (as expected) Patient feedback confirms new process is not upsetting patients ☑	Worthwhile information, retain as permanent change. Easy way to seek patient ideas for areas of improvements.	2

☒, target not achieved; ☑, target achieved; CI, change idea; IP, inpatient; OM, outcome metric; OP, outpatient; PDSA, plan-do-study-act; PM, process metric; SPC, statistical process control; TOE, transoesophageal echocardiography; TTE, transthoracic echocardiography.

Every week, I met with the consultant cardiologist to review the project results, for audit and governance. This helped guide whether further refinement or training was required. I also gathered feedback from the wider team to check their experience.

### PDSA1

Test change idea A (clinical scientist replaces the consultant cardiologist as lead operator) and change idea D (reduce sedation)—which we predicted would impact on different outcome metrics.

We released the consultant cardiologist, pulling in a second cardiac scientist instead, and started weekly lists: capacity (PM1n) increased from six to eight TOEs per month. As shown in table 1, this was successful in reducing waits (OM1in and OM1out) during this cycle, but not sufficiently to meet our targets. We discuss the logic behind the SPC analysis in the Results section and online supplemental appendix C. With the PDSA1 data, there were insufficient data to justify recalculating the SPC limits for OM1in, but there were for OM1out (see figure 2).

10.1136/bmjoq-2023-002268.supp3 Supplementary data

We met the intubation target (0% failures). There were many slots wasted during this cycle (7 of 24=29%, see the Results section for discussion). However, my time was ‘less wasted’ as I could move to inpatient TTE work to help to reduce these waits (light blue on the bottom graph in figure 2).

We also used lower volumes of sedative (PM2) and observed improved intubation outcomes (OM2).

### PDSA2

Test change idea B (clinical scientist review of TOE referrals prior to booking).

I took this on, with guidance from the cardiologist when unsure or needing clarification. I predicted that this would solve inappropriate bookings, reducing wasted slots (PM1w). However, this was more of a challenge than I anticipated, with some referrals requiring more clinical input from the cardiologist.

Disappointingly, PM1w remained at the same rate as the baseline (13%). However, as shown in figure 2, it is lower than during PDSA1 and has allowed greater activity, further reducing the waits during this cycle by inpatients (OM1in) to a mean of 2.1 (working) days and by outpatients (OM1out) to 8.3 (working) days, within our targets of 3 and 14 days.

Now there was sufficient evidence and data to recalculate the SPC for OM1in, and we decided to assign the recalculation point to PDSA1, and enough evidence for provisional recalculation for PM1out once again at PDSA2 (see figure 2).

Note: the other PDSA cycles were relatively small refinements to the process, not impacting on the core results.

### PDSA3

Test change idea C1 (avoid requiring second cardiac clinical scientist).

I took on the task of acquiring the echocardiogram images, so the second clinical scientist was not needed. This avoids the scientist-led TOE service putting more pressure on our other services, in particular TTE.

I predicted that this would not affect the TOE sessions. This proved generally to be the case, but I learnt that I had to ensure everything was in the correct position before starting the procedure and needed to be able to focus on the technical aspects. Importantly, if I needed assistance with the patient (manipulating their head or administering additional sedation if they were not tolerating the TOE probe well), I discovered this put a lot of pressure on the cardiac nurse. We, therefore, requested the support of a nursing assistant to focus on patient safety.

### PDSA4

Test change idea C2 (use a nursing assistant to help with patient management).

This further refinement to session staffing was agreed. Experience (observation) showed this was satisfactory.

### PDSA5

Test change idea D (change patient information leaflet and include satisfaction form).

We made it clear in the leaflet that a clinical scientist rather than a doctor (consultant cardiologist) would generally be performing the TOE procedure, and designed a patient satisfaction questionnaire. This change was not designed to impact operational performance. As expected, we did not pick up any patient unhappiness.

## Results

Our PDSA cycles covered 32 used TOE slots, with 14 of these being under the final process configuration.

Goal 1 was to reduce both inpatient and outpatient waits for TOE. The weekly (rather than fortnightly) lists and increased capacity allowed us to do this. In online supplemental appendix C, we describe how we decided on the SPC analyses of OM1in and OM1out presented in figure 2, as far as possible following methodological guidance.^25 32^ Though we have fewer than the 20 data points recommended to construct the baselines (we have 17 and 19 points, respectively), and would like more data following PDSA2, we are limited by maternity leave and data-system limitations. Nevertheless, we argue in the appendix that our analyses are robust.

The mean waiting times for inpatients (OM1in)are down from 7.7 days to 3.0 days. This meets the internal target we set (3 days); however, this is the mean, so the system is not capable of reliably meeting the target for each patient. The data may suggest some further improvement following PDSA2, but do not yet have enough data to make a robust claim. For outpatients (OM1out), the mean is down from 33.2 days to 8.3 days. Our analysis (online supplemental appendix C) shows that the system has been improved further than it had been in PDSA1, but the limited number of datapoints means that this is a trial or provisional estimate until we have more data (the guidance is 12 data points^25^). This (provisionally) meets the (internal) targets we set ourselves (14 days) and appears (again, provisionally) to be capable of meeting the target reliably (the upper process limit, which shows the range of expected random variation, does not exceed the target).

We noted earlier that rapid diagnosis for inpatients is particularly important, both for diagnosis and for patient flow through the very-congested acute hospital system. We plan to increase our TOE lists from 2 back to 3 slots, so a capacity of 12 per month. We now have the staff capability and capacity to consider running a second list later in the week if inpatient TOEs arise. This would increase capacity by up to a further 12 per month. It would be covered by a consultant cardiologist, or a second fully TOE-trained cardiac scientist. This would also help cover annual/sick leave. This will give us added reliability in coping with demand peaks.

It is disappointing that the wasted TOE slots (PM1w) after the project remained at 13%, as during the baseline. There will always be some ‘lost’ TOE slots, for example, due to DNAs, late-notice cancellations, on-the-day decision by a patient to refuse TOE but agree to TTE in that slot instead. The jump to 29% during PDSA1 was probably random variation. However, now I am running the service, I can use unexpectedly unneeded TOE time to perform inpatient TTE instead.

Review of referrals late in the week (when I’m not in the department) for the next list (Monday morning) is difficult. We may refine idea B, for example, reviewing such cases by video call or (preferably) by the consultant.

We have been very successful on goal 2, reducing intubation failures from 11% to 0%; including cases with complications gives 17% to 0% (OM2, figure 2). I can use lower sedation levels (from a median of 3 mg to 1.5 mg of midazolam) and get better outcomes. Under the old procedures, the cardiologist would sometime use pethidine and metoclopramide in addition to midazolam. In our prechange baseline, there were cases with 5 mg of midazolam and/or additional sedation medications—in three of these, flumazenil had to be used to reverse sedation effects, which is undesirable. Though our sample is fairly small (n=69 with only four failures), and the observational nature of our data means we cannot add the change in operator as a covariate, statistical analysis suggests some tentative evidence of association between sedation volume and intubation success (see online supplemental appendix B).

10.1136/bmjoq-2023-002268.supp2 Supplementary data

Intubation failures are something that we aim to make a rare event. The G-chart is a form of SPC chart designed for situations such as this, using the number of successful cases between successive failure cases as its metric.^25^ This is a tool we plan to use for monitoring in future, and we have constructed one with our current (so far limited) data (see online supplemental figure S4 and accompanying notes).

Goal 3, freeing the consultant cardiologist’s time, has been completely successful. We estimate this is 2 days per month (decreased a little by covering my leave and providing support with assessing some of the referrals.) This high-level assistance of, and even substitution for, consultant-grade doctors’ tasks is the pinnacle of the UK’s Modernising Scientific Careers workforce-development strategy^33^ and its highest-level training component: the doctoral-level HSST programme.^34^ We have done this while increasing capacity and without requiring any additional staff input, other than some nursing assistant time.

Goal 4 was to improve BSE reporting-guideline compliance. Since launching our scientist-led service (PDSA1), all TOE cases are reported to BSE standards and promptly uploaded onto the database. Previously, around 20–30 images would be stored from each imaging; now, we store 60+, giving us reassurance that we have much better coverage. We are satisfied there has been no loss of image quality. A formal quality assurance is in place for any new service audit is in preparation: the imaging lead will review all complex cases with the clinical cardiac scientist including patient history/treatment options to ensure the best possible outcome for the patient. This will also provide continuous training and development for the clinical scientist.

As an added reassurance to patients, as a last PDSA, we updated the information leaflets about the process and staffing; as reassurance to us, we also instigated a patient satisfaction questionnaire. This has shown us that patients are happy with a clinical scientist as TOE operator (rather than a consultant cardiologist). I am also able to better coordinate follow-on TOE investigations arising from our other services, such as the heart valve monitoring clinics. Patients who had experienced the old service mentioned appreciating this continuity of care. All patients have reported a positive experience.

Staff have fed back that the changed service has led to other benefits, including increased utilisation of extended roles, staff motivation, career development and opportunities for more advanced services. This has helped us recruit more staff recently, including locum staff applying for a full-time position mentioning career-development opportunities.

## Lessons and limitations

This project has been very valuable for the cardiac investigations department. In addition to improved performance, directly benefiting patient treatment, inpatient flow and consultant cardiologist capacity, we have an exemplar project to help colleagues to learn about QI—which has already inspired other clinical scientists to become involved in QI and research projects.

Generally, there can be barriers in persuading colleagues and clinicians that clinical scientists are fully capable of extended roles and changing attitudes to our involvement. We were pleasantly surprised, therefore, to encounter no resistance to change nor negativity from any healthcare professional in our trust.

While our new process is ‘lean’ in making the most of staff skills,^16^ it is important to remember that the core of lean thinking is making workflows fast and efficient rather than minimising staffing—staff development is a key part of the Toyota ethos.^35^ There will be occasions when we do want a second clinical scientist present for training and role enrichment, which would release the nursing assistant for professional development

As my first QI project, there were many techniques to get to grips with, but I found the overall process was easier than I feared because of the alignment of the project with my commitment to patient care and passion for scientist-led services. Particularly for a first QI project, I feel this close alignment is important for motivation. The 4N technique proved especially useful in engaging colleagues.

I found the detailed data analysis challenging, pulling information together from many sources. In the pressured NHS clinical-delivery environment, there are always other priorities which can seem more important than data collection and analysis. However, I see the value, and data will continue to be the foundation of my future projects. In retrospect, larger datasets would be desirable for each phase. There is a trade-off between this and the desire (or imperative) to get on and make changes to benefit a pressured service and current patients.

Similarly, we noted that the data available for SPC are a little less than recommended, particularly for the technique we used (XmR). The value, and easier analysis, from longer a baseline and more datapoints between and after PDSA changes are evident, and an important lesson. Though again we feel the trade-off between prompt action and delay for further analysis.

Our data do support the view that intubation can benefit from lower sedation, though our small sample makes us very cautious about the statistical analysis (online supplemental appendix B). Further, the new design has changed both sedation practice and TOE operator. This natural experiment and ethical considerations therefore make it impossible to separate the individual impact of sedation.

Potential confounds to claims for performance improvement in a system like this could have been reduced demand or increased staffing levels. Demand for echocardiography remained high, and the large waiting list and long waiting times (at NWAFT along with many other hospital trusts^5^) were noted at the start of this paper. Staffing for the service was not increased during the project—in fact a key benefit was the reduced demand on high-value clinical staff capacity.

## Conclusion

This QI project used the MfI to explore and refine change ideas to improve our TOE service by redesigning the clinical scientist role to increase capacity and so greatly reduce patient waits. This project has been successful, achieving its aim, and is proving to be safe and efficient with increased activity. Additional benefits include freeing considerable consultant cardiologist time for higher-value work and providing an enriched role for me as a clinical scientist (on the track to becoming a consultant clinical scientist through the HSST programme). We intend to continue developing our TOE service, including presenting TOE cases in multidisciplinary team meetings to assist with clinical decision-making about treatment pathways. The experience and results also encourage us to extend roles elsewhere in our department.

## Data Availability

Data are available upon reasonable request.
