# Supplementary material for: Clinical-scientist-led transoesophageal echocardiography (TOE): using extended roles to improve the service
Source: BMJ Open Qual. 2023 Sep 25;12(3):e002268. doi: 10.1136/bmjoq-2023-002268 (PMC10533795; doi:10.1136/bmjoq-2023-002268)
Supplement: Supplementary data [file bmjoq-2023-002268supp004.pdf]

Figure S1 : Fishbone Diagram

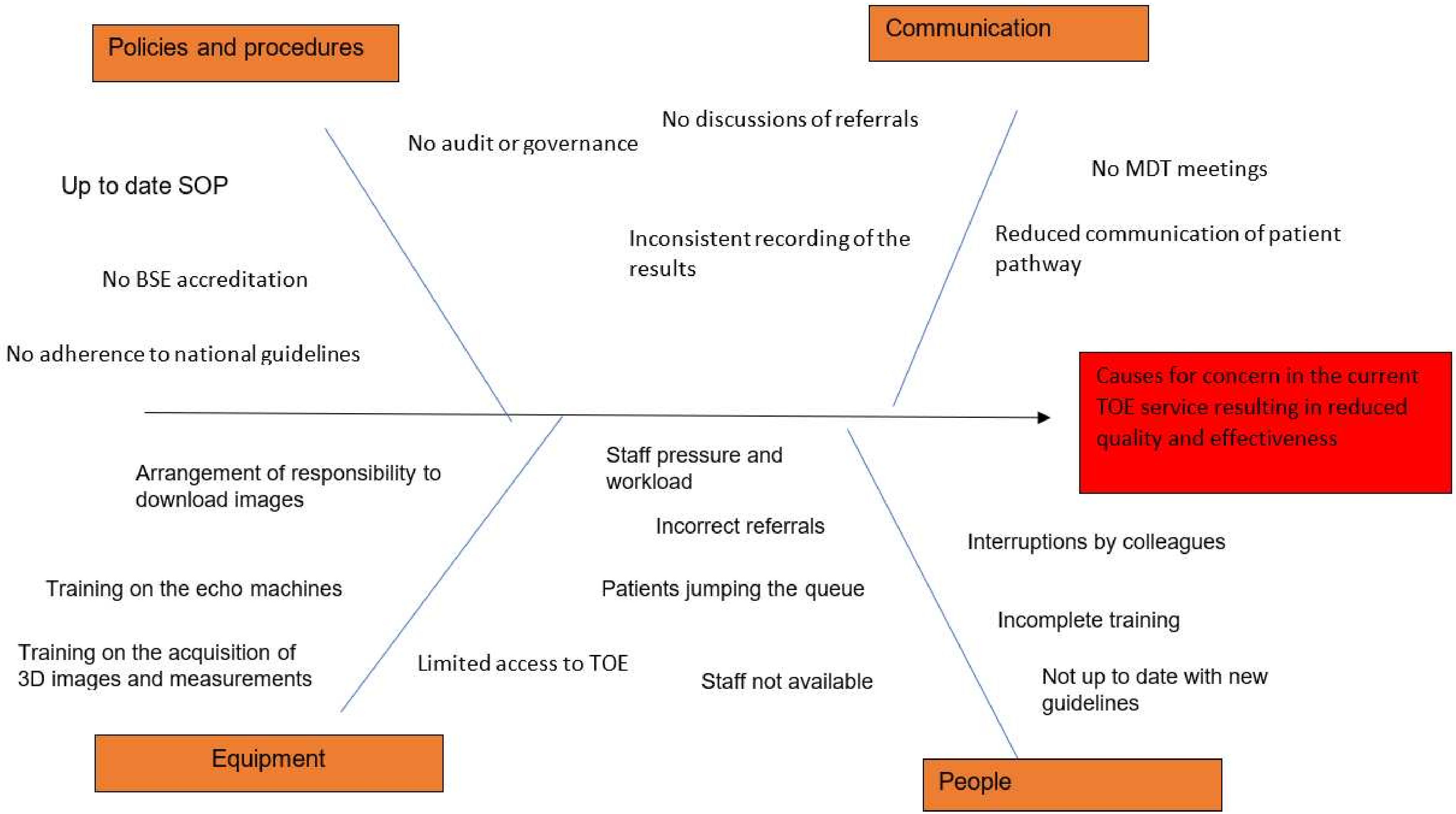

BMJ Publishing Group Limited (BMJ) disclaims all liability and responsibility arising from any reliance placed on this supplemental material which has been supplied by the author(s)

Figure S2 : 4N Chart demonstrating that staff were frustrated with the wasted time during a TOE and the increased patient waiting times (yellow = areas of focus for this QI project, IP = inpatient)

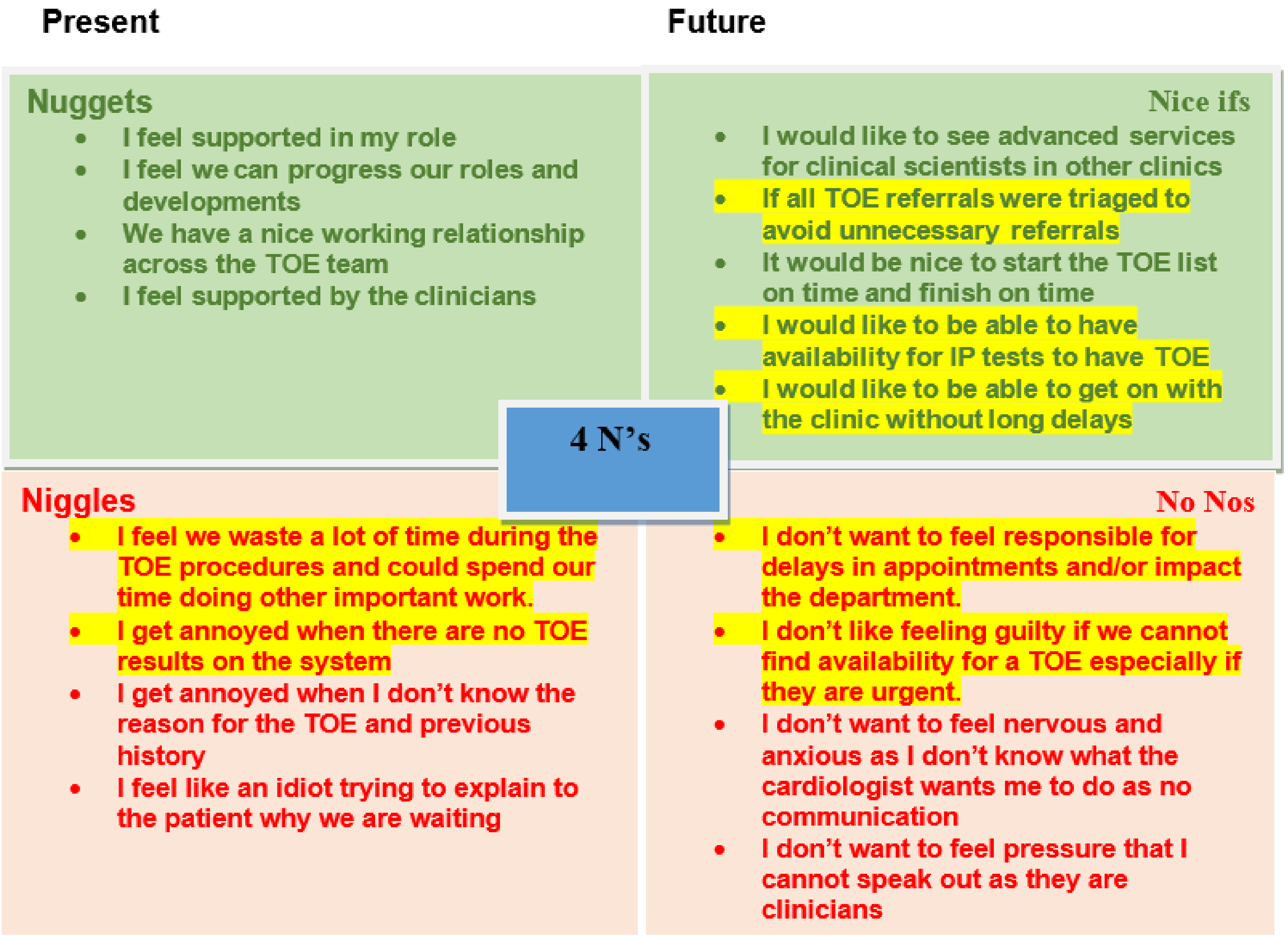

Figure S3: Driver Diagram

also locating the metrics (OM = outcome metric, PM = process metric, BSE = British Society of Echocardiography)

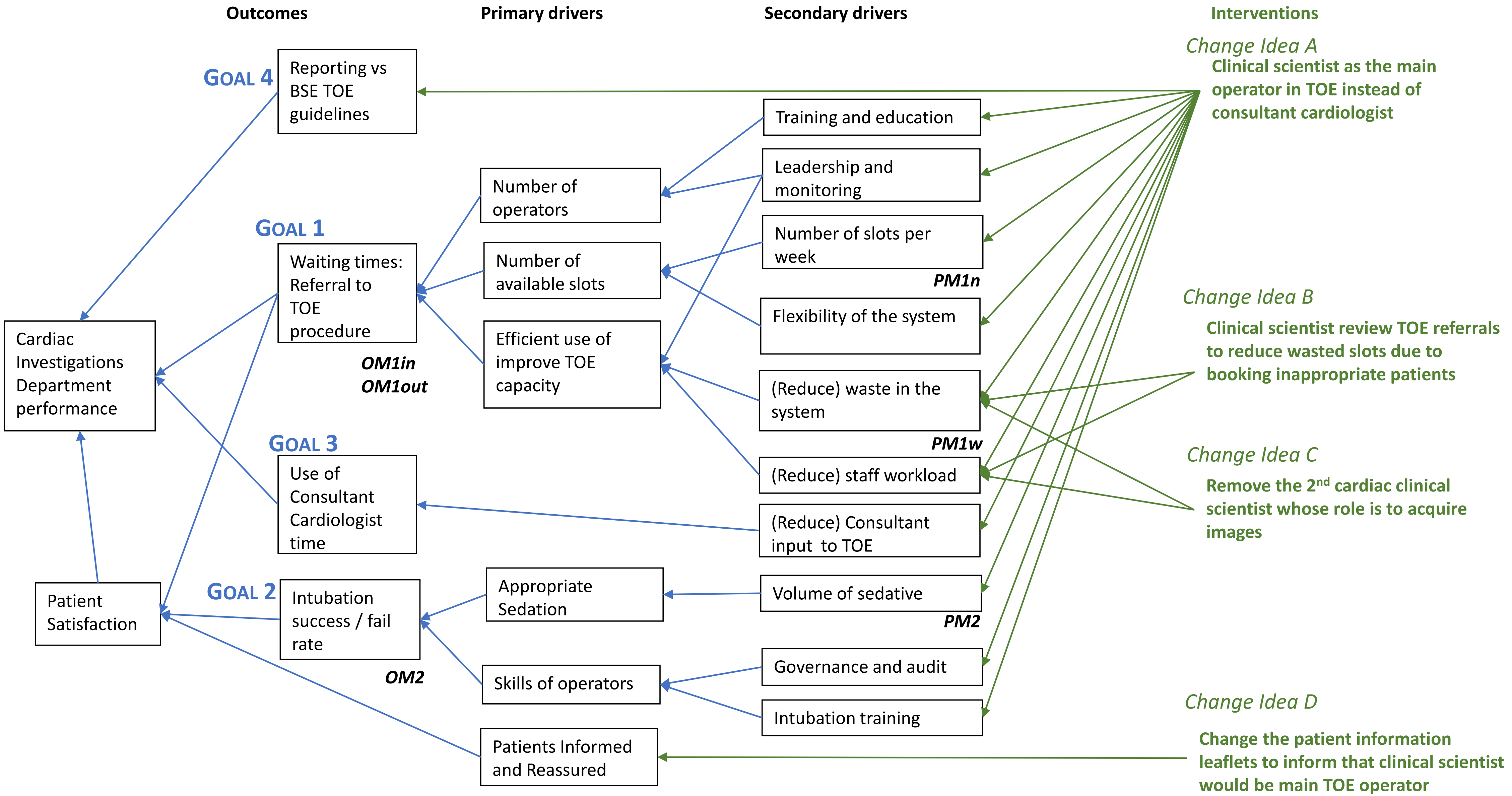

Table S1 : Niggle-o-gram for staff satisfaction: niggles experienced by staff members involved with the prior TOE service (yellow = agreed priorities)

Niggle	Incidence (0-9)	Impact (0-9)	Influence (0-9)	Code (0-9)	Priority
Takes too long to perform a TOE with all the delays	8	9	9	8-9-9	3
Wasted time waiting for the cardiologist during the TOE	8	9	5	8-9-5	7
No communication between operator (cardiologist) and the clinical scientist and cardiac nurse.	7	7	4	7-7-4	8
------------------ Becomes irrelevant with change to be scientist-led (PDSA A) ------------------					
No report on the system so when the patient comes back for a TTE, there is no results for guidance.	9	9	0	9-9-0	6
Trying to find slots and no flexibility on appointments	8	8	8	8-8-8	2
Long waiting times for a TOE which delays patients management	8	8	8	8-8-8	1
Limited progression in cardiac science	8	8	8	8-8-8	4
Limited training for progression	8	8	8	8-8-8	5

Table S2 Potential barriers to the QI project and proposed actions to overcome them

Potential barriers	Details	Proposed actions
Lack of support	- No support from the trust<br>No governance approval<br>No mentor for training	- Propose the benefits to the trust and governance<br>Prepare a letter for governance<br>Provide evidence of other organizations which have the process in place
Lack of understanding	- Other healthcare professionals<br>Not a clinician performing the test	- Explain the needs of improving the services of advanced imaging to support the pressures and workload.<br>More patients are undergoing valvular intervention who require advanced imaging prior to surgery.
Lack of staff engagement	- Lack of knowledge and understanding of the vision<br>No involved in the process<br>Don't want to be involved<br>Don't want to progress	- Share the vision from the onset<br>Embedding QI within the department<br>Show how this will benefit the team
Staff shortages and workload	- Consultants' ability to provide support and training<br>Clinical scientist shortage in main TTE to release them for advanced training<br>Time for training and teaching	- Long term gain would be to reduce unnecessary referrals.<br>Reduce number of TTE and go straight to TOE
Lack of training and education	- Lack of confidence in competence in ability<br>Ensure enough numbers for competence	- Could be released to other trusts for the training<br>Be involved with every consultant TOE list to improve confidence
Lack of supervision and leadership	- Clinicians maybe reluctant to relinquish advanced procedural skills, perhaps concerned about the consequences on their own individual practice.	- Propose the expansion of services which require multiple operators to reduce pressures<br>Expanding roles will help deliver increased imaging demand
Lack of evidence	- Lack of evidence such as policy and procedure	- Demonstrate to the organization the need for the service and its success at other trusts.<br>The cardiology workforce, GIRFT report and NHS Long term plan explain the need for advanced training in healthcare scientists.
Reduced opportunities for cardiology trainees	- Training clinical scientists may reduce training opportunities for cardiology doctors<br>Although with time, clinical scientist will be involved in the training of medical juniors as well as other clinical scientists.	- Clinical scientist train cardiology trainees in TTE therefore could eventually train in TOE<br>Consultant cardiologist lists could be training for cardiology trainees especially for the intubation.

Figure S4: SPC G-Chart of Cases between *Complication* or *Failure* Cases

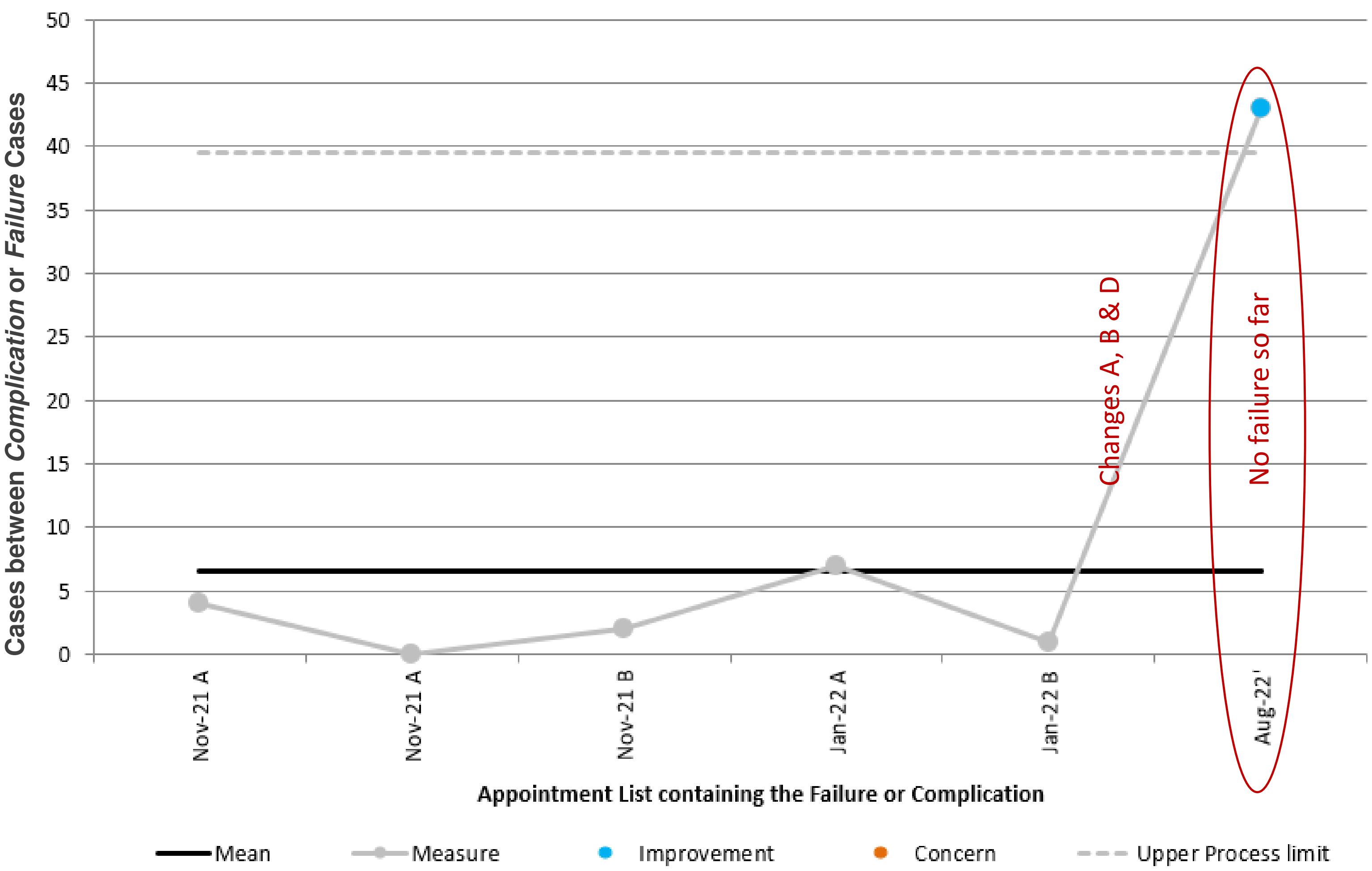

The A and B are the first and second of the two lists per month (prior to the change to the scientist led service).

G-chart format SPC chart (see Provost and Murray, 2022, pp. 198-201 for technical details), using the NHS Excel template.

This format is designed for relatively rare events. Pre-change these were not very rare, but appear to have become so post-change.

The first 5 (grey) points are our complication or failure events shown on Fig 2. The metric is the number of cases between these events, so the first of our events in Fig 2 has censoring before it (the previous complication or failure event off to the left of Fig 2's PM2 graph so is not included here).

The last (**blue**) datapoint is the end of our data – with no complication or failure *so far.*

The horizontal line, the Centre Line (CL), is the 'theoretical median'. If *Gbar* is the mean of the number of cases between events, then:

theoretical median = ln(2) × *Gbar*

The template shows the SPC-style Upper Limit (UL) , which works out to be 39.46 cases, suggesting special cause (improvement) has been achieved.

However, we have very few data points so cannot establish a strong baseline. Nevertheless, any special cause is "a valid indication of a process with important special causes") (Provost and Murray, 2022, p.276).
Alternatively, we might ignore this process limit and treat it like an early stage run-chart analysis. Then, as with special cause on SPC, this last 'so far' data point might be considered 'astronomical' (Rule 4) even with these limited data "because it is a visual analysis not dependent on a median [and so] can be applied at any time based on the user's degree of belief" (*op. cit.* p.100).
