# Supplementary material for: Clinical-scientist-led transoesophageal echocardiography (TOE): using extended roles to improve the service
Source: BMJ Open Qual. 2023 Sep 25;12(3):e002268. doi: 10.1136/bmjoq-2023-002268 (PMC10533795; doi:10.1136/bmjoq-2023-002268)
Supplement: Supplementary data [file bmjoq-2023-002268supp001.pdf]

Appendix : Notes on training requirements for clinical cardiac scientists in TOE

Nikki Kaye, 2023

The clinical cardiac scientist will need a mentor to support them through the process of TOE training. Almost inevitably this will be a clinician, usually a cardiologist. The mentor should be accomplished in TOE, ideally with accreditation from the British Society of Echocardiography (BSE) or equivalent.

Training BSE accreditation

The BSE provides an accreditation process designed to set standards for, and test competence in, performing and reporting TOE studies. Their accreditation process is run as a service for practising echocardiographers. Though it is not compulsory or a regulatory requirement, it is recognised nationally and internationally as a mark of competence.

The BSE suggests that continuing education must be provided for TOE operators, and each operator must perform or directly supervise at least 50 studies per annum. It is the responsibility of the operator to monitor and keep evidence of continuing education.

BSE accreditation and training requirements

- A centre must have a designated Head of TOE who must have the BSE/ACTA or EAE TOE accreditation. They will be responsible for appropriate training for other operators.
- The Head of TOE should allow a reasonable length of time (for example 2-3 years) to achieve TOE accreditation, or will be given the opportunity to transfer the TOE lead role to a suitably experienced and TOE-accredited colleague.
- All primary operators must have, or be working towards, BSE/ACTA TOE accreditation.
- The accreditation process requires the candidate to submit a log-book and pass a written examination within a continuous 24-month period.
- Outpatient TOE studies must have an operator with appropriate training and with support of a nurse experienced in managing airways.
- For the full TOE accreditation process, see the BSE TOE accreditation pack: www.bsecho.org/Public/Accreditation/Personal-accred/Personal-accreditation.aspx

Maintaining BSE accreditation and appropriate skills

- An operator needs regular protected lists (at least 2-3 per month)
- Continuous membership of the BSE
- Re-accreditation is required every 5 years, examining evidence of continuing clinical activity, distance learning and attendance at courses and conferences.

Kaye N, *et al. BMJ Open Qual* 2023; 12:e002268. doi: 10.1136/bmjoq-2023-002268

Additional training and skills required

All clinical cardiac scientists should complete additional training outside the BSE TOE accreditation to ensure competency and to ensure that their job descriptions are reviewed to reflect their new responsibilities.

A suggested training plan for additional training for the clinical scientist is outlined below. This assumes a significant level of experience in regularly consultant led TOE and competency with additional. Required skills.

1. Observation - Regular observation in consultant led TOE is required to understand the process of the procedure and increase the knowledge of the additional anatomy seen on TOE. In addition, regular observation in reporting with consultant
2. Practical under direct supervision
3. Intubation skills
4. Informed consent
5. Cannulation training

Table 1: Summary of the requirements for scientist-led TOE service.

Task
Clinical Scientist must obtain:
➢ HCPC registration<br>➢ BSE TOE accreditation<br>- Case studies (part of BSE accreditation)<br>- Exam (part of BSE accreditation)
Advanced training for clinical scientist includes:
➢ Regular observation in consultant-led TOE – understand the process and increase knowledge of the additional anatomy seen in TOE.<br>➢ Supervised practical skills (minimum 12-18 months)<br>➢ Intubation training and manipulation (12-18 months)<br>➢ Informed consent<br>➢ Cannulation training<br>➢ Have a patient specific directive (PSD) in place for conscious sedation if the scientist is responsible for sedation.<br>➢ Continuous development and training
Cardiac Nurse to lead sedation:
➢ Regular observation in consultant led TOE<br>➢ Supervised practical skills of safe sedation<br>➢ Good communication with the clinical scientist<br>➢ Be ALS trained (or ISL if the clinical scientist is ALS trained)
Organisation
➢ Approval from governance<br>➢ Sign off Patient Group Directive (PGD) if the cardiac nurse is responsible for safe sedation.<br>➢ Standard Operating Procedure (SOP)
Department

- Clinical governance
- Audit
- Quality assurance (review images and complex cases)

Quality assurance and audit

Formal audit process must be in place. In centres with several TOE operators interval re-reporting of studies and/or comparison against surgical results is adequate.

A formal quality assurance system should be in place including: weekly review meetings, regular process audits consisting of blind over reading of selected studies to ensure compliance with departmental minimum standards, regular audit projects to check consistency of interpretation such as requiring all team members to report a set of images, outcome audit against other modalities or surgical results. Regular meetings, ideally weekly, must be held to review unusual, challenging or otherwise difficult cases. There must be established processes issuing appropriately revised reports as a result of multi-disciplinary team discussions.

Kaye N, *et al. BMJ Open Qual* 2023; 12:e002268. doi: 10.1136/bmjoq-2023-002268
