# Supplementary material for: Clinical-scientist-led transoesophageal echocardiography (TOE): using extended roles to improve the service
Source: BMJ Open Qual. 2023 Sep 25;12(3):e002268. doi: 10.1136/bmjoq-2023-002268 (PMC10533795; doi:10.1136/bmjoq-2023-002268)
Supplement: Supplementary data [file bmjoq-2023-002268supp003.pdf]

Appendix C Consideration of SPC Analyses

Considering first OM1in – the inpatient waiting time from Referral to Receiving the TOE procedure (in days):

- We have **17** pre-change (baseline) datapoints, fewer than **20**-25 (Mohammed et al, 2008) or **20**-30 datapoints (Provost and Murray, 2022) recommended for XmR charts. This is unfortunate, but we cannot access more data retrospectively. It is a limitation and a learning point.

- If there are fewer than 12 datapoints a run chart should be used instead (Provost and Murray, 2022), but we have more than this.
- With fewer than 20 datapoints for a baseline we should regard the SPC centre line and process limits as 'soft' or 'provisional' (Mohammed et al, 2008) or '**trial limits**' (Provost and Murray, 2022); this is more important for the XmR (as we are using) than for other SPC formats (Provost and Murray, 2022, pp.141-143, 165). It is recommended that a more robust centre line and process limits are recalculated once 20 points are available (op. cit.).

- Provost and Murray (2022, p.142) give an example of progressing from a run chart (with n=11 points), to an individuals (XmR) SPC chart with trial limits that can be frozen and extended (when k=12 is reached), and then repeating for robust limits (when k=20 is reached).
- Applying this to our data, we have trial limits calculated from the pre-change data (k=17) and extended (purple) (left) and this repeated taking in 3 post-change points to reach k=20 (right).

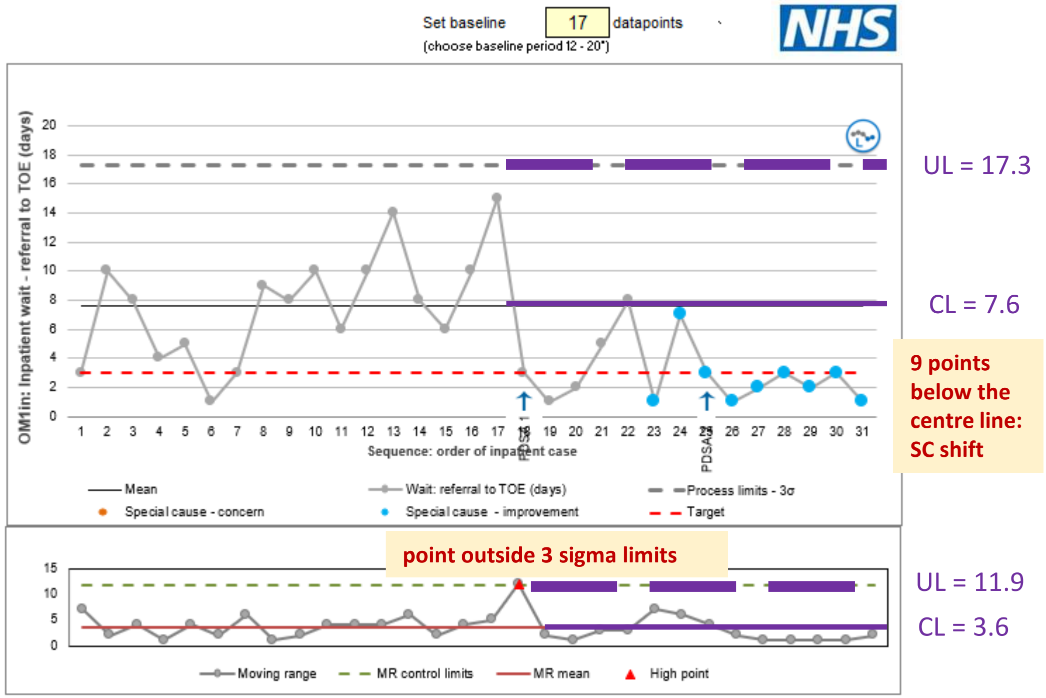

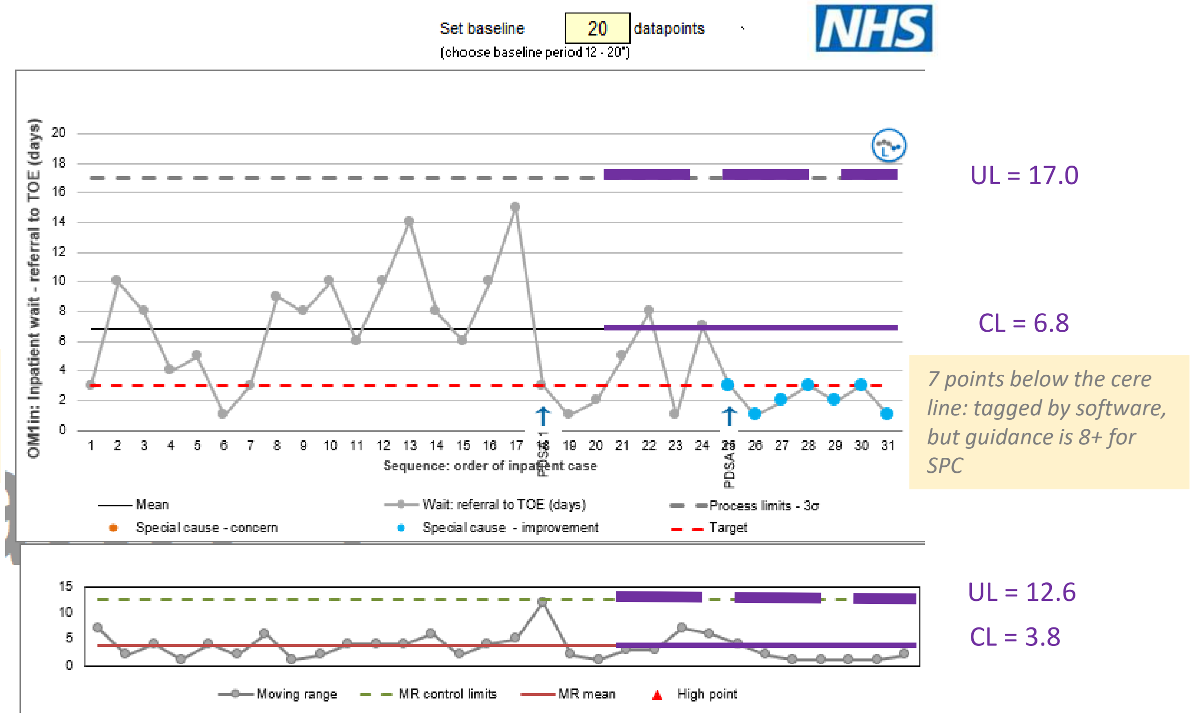

- We observe, firstly, that the 3 extra points **make very little difference** to the centre line (mean) and limits (3 sigma).

- Secondly, it is important to note that whilst trial limits "may not be useful" especially for XmR (op. cit., p.143), "**for any number of subgroups [i.e., for any number of datapoints] a special cause signal will be a valid indication of a process with important special causes**"; "So, if we have used fewer that [*sic*] the advised number of subgroups to create limits and we still detect special cause we do not have to worry about "false" special cause signals." (Provost and Murray, 2022, p.276).

- The left graph highlights Rule 1 beyond the limits special case on the mR chart, which should be looked at first (Provost and Murray, 2022, p.281). The right graph highlights only Rule 2 shift special cause on the X chart – though note that the NHS template tool will only trigger on 6+ or 7+ points one side of the CL rather than the 8+ usually recommended (op. cit, p.135), there are 7 on the right graph but 9 on the left.

- On possible conclusion (particularly if the baseline or intervention were unclear) would be to focus on the right-hand graph (and discount the <8 points of shift) and conclude that there had been no change.
- Instead, we would argue for lefthand graph
  - That the similarity between the limits of the two charts mean that the short baseline (k=17), left, *is* useful (the quote above was "*may* not be useful"; our emphasis added), so focus on the left graph.
- This lefthand graph triggers **Circumstance 3 of the guidance on when limits should be recalculated**: "When changes tested have resulted in desirable special cause (improvement) on a Shewhart chart… In this case the centreline and limits should then be calculated for the new process." (Provost and Murray, 2022, p.145).
- This is also discussed (op. cit, pp.288-289) as the alternative causal strategy, to evaluate the impact of a known intervention [without automatically recalculating the limits at known changes], and guided by subject matter [domain] experts (op. cit, pp.273, 275).
- We then have to decide on when the change occurred. "Use special cause signals relative to the extended limits to decide if changes made after the baseline data have an important effect on the measure. If the pattern of data points suggests a change in performance, recalculate new limits to reflect the new process."

- As the baseline data has < 20 datapoints, we might have fallen back on **run chart analysis** (Provost and Murray, 2022, pp.90+).
  - A baseline median can be built from any number of datapoints, but to use the probability-based rules for detecting change 10+ is recommended. We have that here (k=17).
  - Doing this we observe a strong signal of shift (the point *on* the median is ignored) - *all* counted datapoints post baseline are below the median: 13, far in excess of the 6 required in the guidance.

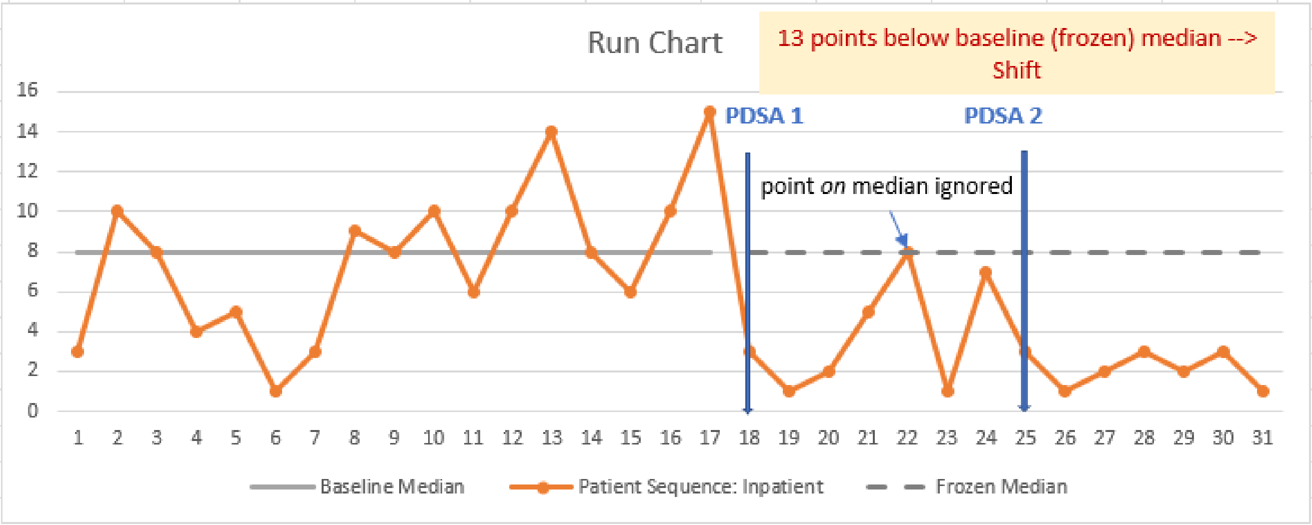

- Considering the lefthand SPC and the run chart, we argue that the best interpretation of the data is that a shift occurred – accounting for
    - the mR SC at the start of PDSA1
    - The pronounced shift on the run chart from the start of PDSA1
    - The SPC shift signal triggered by the last 9 of the PDSA1 and 2 datapoints

- “Use special cause signals relative to the extended limits to decide if changes made after the baseline data have an important effect on the measure. If the pattern of data points suggests a change in performance, recalculate new limits to reflect the new process.” (Provost and Murray, 2022, p.289).

- We thus recalculate at the start of PDSA1, which shows stable behaviour before and after, and the mR SC is filtered out as it is at the point of behaviour change.
- After the recalculation we have 14 datapoints, satisfying than the 12+ recommended for robust new process performance measures (Provost and Murray, 2022, p.276).

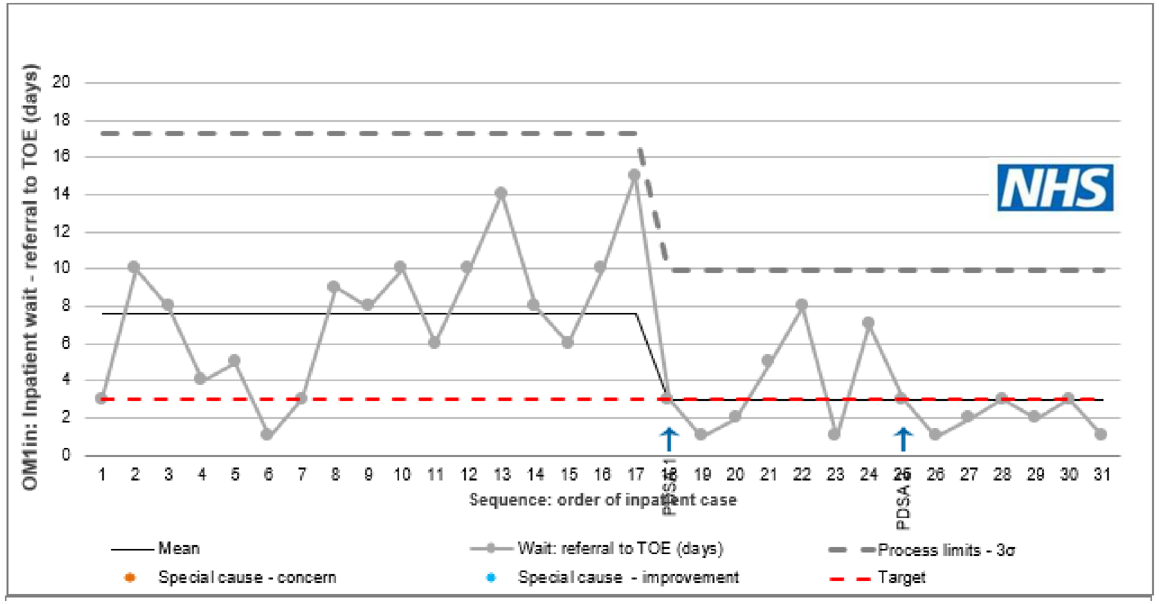

CL = 3.0

- We have no evidence from the SPC of material change in behaviour in PDSA2, though note that the behaviour shows early visual signs of improvement (shorter times and les variation).

- **This is the chart we use in Figure 2 for OM1in.**

Repeating this for OM1out – the outpatient waiting time from Referral to Receiving the TOE procedure (in days):

- We have **19** pre-change (baseline) datapoints, only one fewer than the recommended minimum of 20, again unfortunate, but we cannot access more data retrospectively.

- The centre line and limits are little different if we use the first 20 datapoints to construct our centre line and limits rather than 20.
- In addition, the SPC diagnosis is identical: a very definitive shift around the time of the changes introduced in PDSA1.

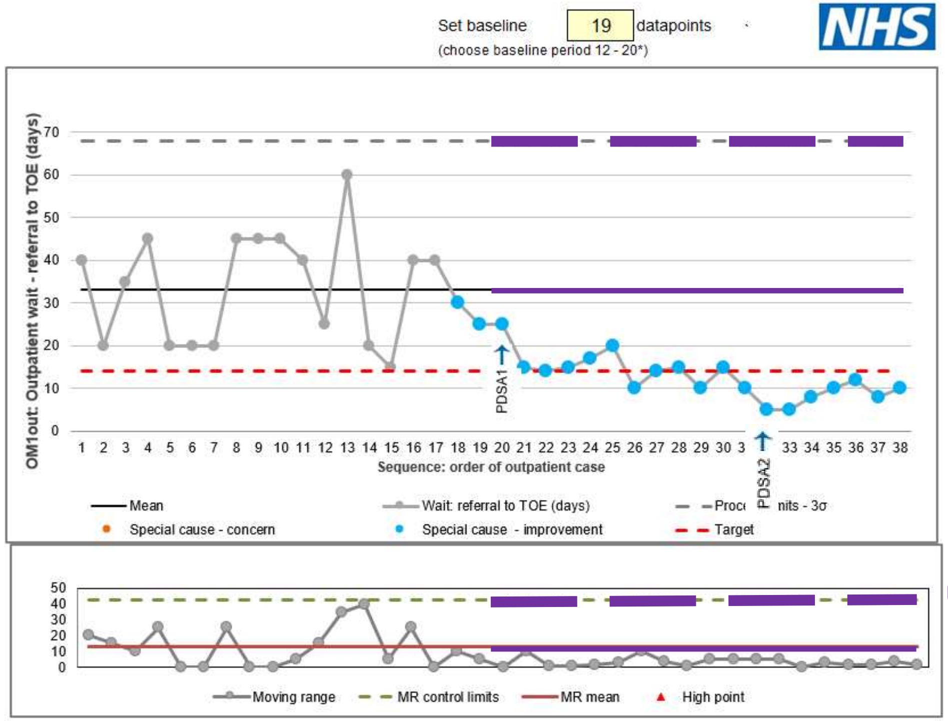

UL = 67. 9

CL = 33.2

UL = 42.7

CL = 13.1

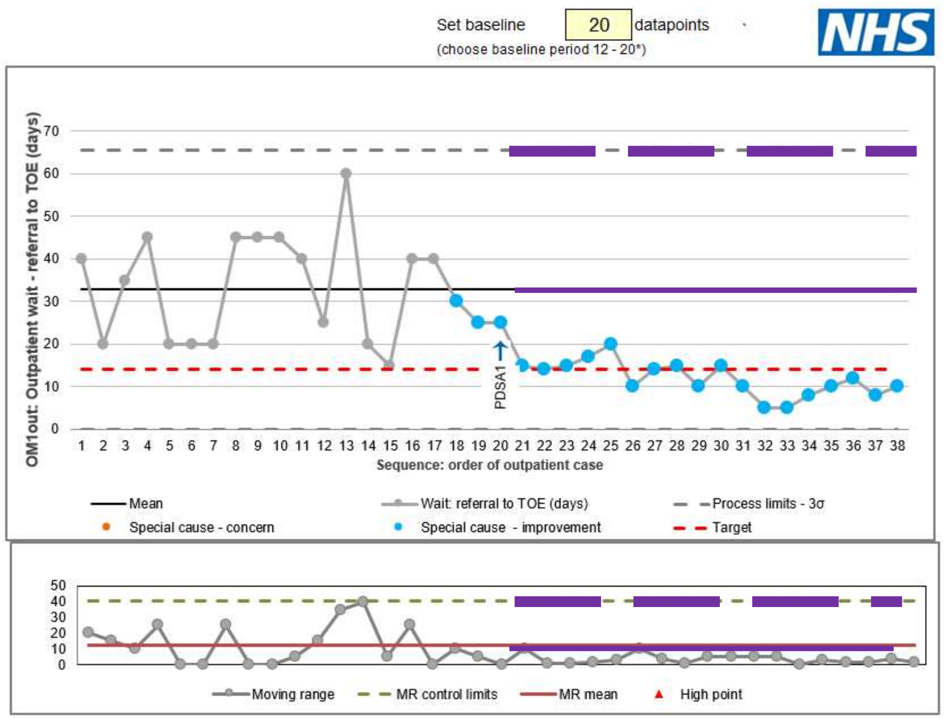

UL = 65.7

CL = 32.8

UL = 40.4

CL = 12.4

- There is strong evidence to recalculate at PDSA1.
- We have 12 datapoints from PDSA1, so sufficient to recalculate, freeze and extend again.

- We now see 8 points (the last 8) below the new centre line.
- Remembering that, if we see a special cause, it is always a valid signal, we argue we are justified to recalculate again at PDSA2

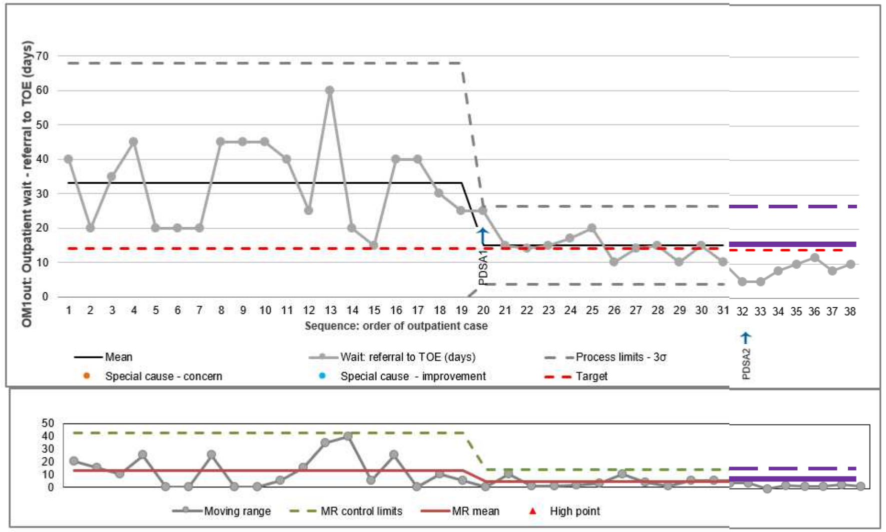

CL = 15.0 (both mean and median = 15)

8 points below the centre line: SC shift

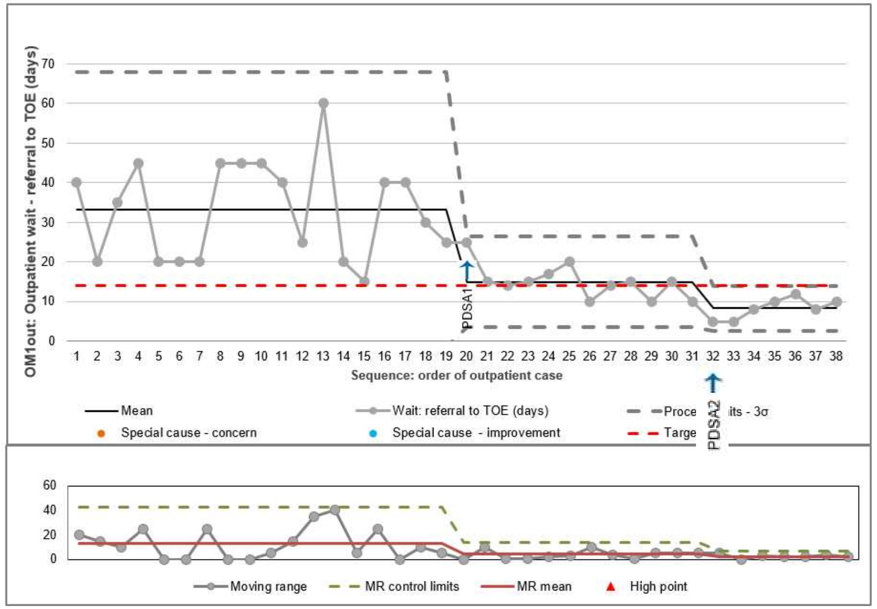

CL = 8.3

- Now (right) we see no special cause.
- This gives us new trial or soft centre line and limits to estimate the performance of the process.
- **This is the chart we use in Figure 2 for OM1out.**

- This gives us a mean waiting time of 8.29 days, or 8 if we use the median for that last segment of data (PDSA2) – a temporary run chart on the SPC until we have 12 datapoints (Provost and Murray, 2022, p.276).

- We could then argue that it would be useful to go back to OM1in and recalculate at PDSA2 also to reflect this new phase consistently across the 'family' of this pair of metrics (op.cit., p.277), but we have decided to be conservative and leave the recalculation only at PDSA1.
