# Supplementary material for: Clinical-scientist-led transoesophageal echocardiography (TOE): using extended roles to improve the service
Source: BMJ Open Qual. 2023 Sep 25;12(3):e002268. doi: 10.1136/bmjoq-2023-002268 (PMC10533795; doi:10.1136/bmjoq-2023-002268)
Supplement: Supplementary data [file bmjoq-2023-002268supp002.pdf]

Appendix B – Statistical Analysis

The QI project involved changing from a 'Pre' regime, under which the Consultant Cardiologist decided the volume of sedative and operated the TOE probe, to the 'Post' regime, under which the Clinical Scientist took over both tasks, so there are two independent variables **Sedation** and **Operator**. The outcome is an intubation Success or Failure.

Figure 2 in the main paper shows both the volume of sedative and the outcome (intubation success of failure) across the project.

In this appendix we apply some further statistical tools, but with great caution since i) we have a limited amount of data and ii) it is observation data (from a natural rather than designed experiment).

Contingency Table

Sedation	Pre			Post			Total	
(mg)	Success	Failure	Total	Success	Failure	Total	Success	Failure
0.5	0	0	0	1	0	1	1	0
1.0	3	0	3	9	0	9	12	0
1.5	5	0	5	8	0	8	13	0
2.0	5	0	5	10	0	10	15	0
2.5	0	0	0	1	0	1	1	0
3.0	12	0	12	3	0	3	15	0
3.5	1	0	1	0	0	0	1	0
4.0	6	1	7	0	0	0	6	1
4.5	0	0	0	0	0	0	0	0
5.0	1	3	4	0	0	0	1	3
Total	33	4	37	32	0	32	65	4

Table B1 : Outcome by Sedation volume and Regime (Pre = Consultant, Post = Clinical Scientist)

The data are too sparse to look at both Operator and Sedation. We can though consider the association of the Sedation with the outcome, *assuming* no effect of Operator and no systematic difference between the patients under the two regimes.

Impact of Sedation

Note: Sedation was administered in units of 0.5mg. The X-Y graph below uses jittering (adding a small amount of random noise to the data display) to avoid overprinting; the histogram uses 'dodging' (showing the split data offset) also to avoid overprinting.

Analysis of Volume of Sedation

We can see that

- the Sedation *does* tend to be different by regime (Pre vs Post)

Kaye N, *et al. BMJ Open Qual* 2023; 12:e002268. doi: 10.1136/bmjoq-2023-002268

in Table B1 and the histogram in B2 (and the Mann-Whitney test give $p < .005$) – under the Post regime less Sedation tended to be administered (by the Clinical Scientist) than in the Pre regime (by the Consultant). The medians are 1.5mg vs 3.0mg.

Analysis of Outcome by Volume of Sedation

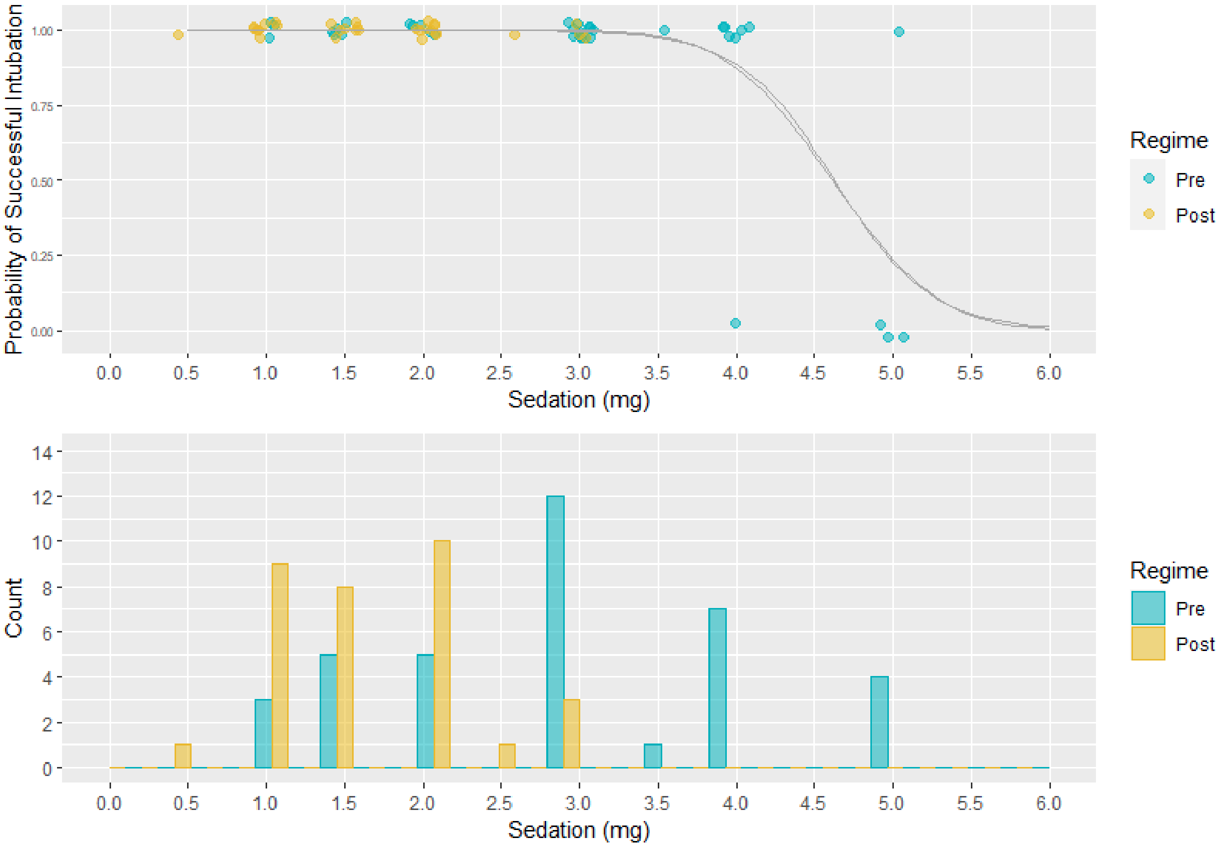

Figure B1 upper: scatterplot with probit and logit fitted curves; lower: histogram (note: data are jittered or dodged around the actual values which are multiples of 0.5mg)

Attempting binary regression to model the outcome (Success / Fail) from the Sedation, by fitting the logit model (logistic regression):

$$P(Success) = \frac{1}{1 + e^{-(b_0 + b_1 Sedation)}}$$

And the probit model:

$$P(Success) = \Phi(b_0 + b_1 Sedation)$$

where Φ is the cumulative distribution function of the standard normal distribution.

Give almost identical results, as shown in Figure B1 (upper), with

- Sedation being statistically significant ($p=.015$) in both.

Using cross-validation[1] (with k=2 folds, because the dataset is fairly small, and 20 repeats), using the caret package,[2] gives Cohen's κ (kappa) of around .72 with standard deviations of around .17 (Monte Carlo techniques are used, so results differ slightly each time the procedure is run).

κ is a measure of correlation between raters (e.g. models vs observed) robust to imbalanced groups. Cohen suggested values of .61 to .80 indicate substantial agreement; a more recent suggestion is that .60 to .79 be regarded as moderate agreement.[3]

- We can therefor regard the logit or probit models as moderately robust (even with this small amount of data)

We might typically locate Sedation ≤ 4.5mg (i.e. as a threshold (P(Success) remains above 50%) but, given the impact on the patient and waste of resources from a failure (abandonment of the procedure), we might want to suggest a threshold with a much higher probability of success.

Experimenting with a conditional inference classification and regression tree,[4] suggests a threshold of Sedation ≤ 3.5mg, splitting our 58 datapoints with that level of Sedation and 100% success, versus the 11 above where success was 7/11 = 64%.

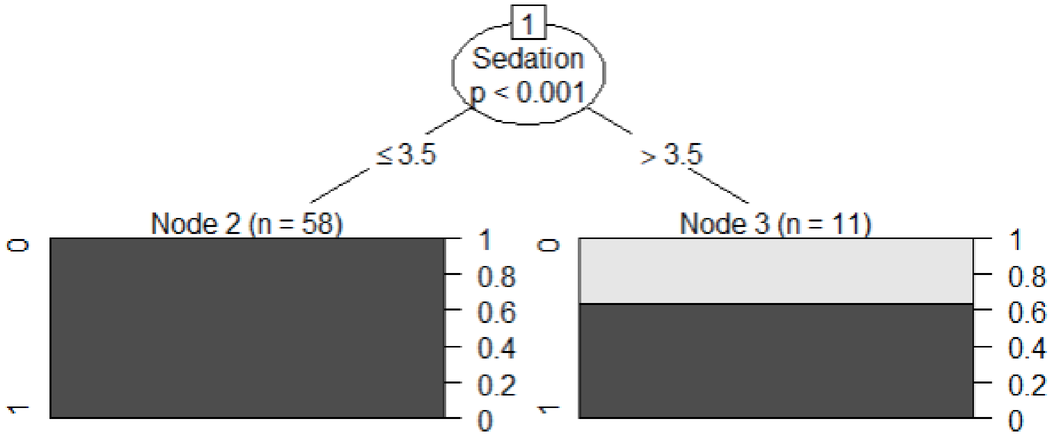

These analyses suggest this small study

- does support the hypothesis that Sedation volume is indeed associated with intubation success,
- and Sedation > 3.5mg should be regarded with caution.

References

1. James G, Witten D, Hastie T, et al. *An introduction to statistical learning - with applications in R*. 6th corrected printing ed. New York: Springer, 2015.

2. Kuhn M, Wing J, Weston S, et al. caret: Classification and Regression Training version 6.0-94. 2023. CRAN. https://cran.r-project.org/web/packages/caret/caret.pdf. (accessed 30 April 2023).
3. McHugh ML. Interrater reliability: the kappa statistic. *Biochem Med (Zagreb)* 2012;**22**:276-82. doi:10.11613/BM.2012.031
4. Hothorn T, Zeileis A. partykit: A modular toolkit for recursive partytioning in R. *The Journal of Machine Learning Research* 2015;**16**(1):3905-09.
